# Cassini-oval description of atomic binding: New insights into the quantitative relationship between hardness coefficient and bond energy energy

**DOI:** 10.1371/journal.pone.0303311

**Published:** 2024-05-16

**Authors:** Weicheng Zeng

**Affiliations:** Xiamen Medicine Research Institute, Xiamen, Fujian, China; Al Mansour University College-Baghdad-Iraq, IRAQ

## Abstract

In this paper, the research hypothesis of conservation of hardness coefficient is put forward, and a mathematical formula for describing and analyzing the relationship between hardness coefficient and bond energy is established in this model. The binding process of two heteronuclear atoms can be represented by Cassini oval in dynamic form, every molecular state corresponds to one of these graphs; then the critical phenomena of molecular deformation are discovered and the calculated potential energy at the critical point is consistent with the experimental dissociation energy of molecules (R is 0.99999, *P* < 0.0001).

## Introduction

Upon the formation of a molecule, the electron transfer between the atoms must be accompanied by some changes in hardness and shape of the atom, this realization sparked a new understanding of the atomic properties and the atomic deformation potential model has been proposed in the Letter [1], there is a constraint relationship between hardness and deformation of atomic particles, each atom in a homonuclear diatomic molecule is described by three parameters *a*, *b* and *ζ*_0_ and the atoms are the most stable when the value *aζ*_0_/*b* is constant, *a*/*b* can be regarded as the atomic hardness coefficient. In homonuclear diatomic molecules, the proposed atomic hardness coefficient is related to molecular potential energy; In heteronuclear diatomic molecules, if the hardness coefficient is still an indispensable factor of molecular potential energy function, how the hardness coefficient evolves with the chemical environment is a question that needs to be explored.

There are many theoretical models that can be used to calculate atomic hardness [2–6], but the relationship between atomic hardness coefficient and molecular potential energy is still to be determined. The main innovations of this paper include the following parts. Firstly, a new hypothesis about conservation of hardness coefficient is suggested. It is an intuitive idea that the hardness coefficient of one atom in the molecule decreases, which is equal to the hardness coefficient of another atom increases; Secondly, discovering that the molecular state can be described by Cassini oval in dynamic form [7, 8] and the molecular deformation potential corresponds to the shape of Cassini ovals. In particular, this model uses Newton mechanics to calculate the bond energy of molecules, rather than using wave mechanics as scholars typically do. [9–11].

## Materials and methods

### 1. Derivation of potential energy

Like the homonuclear diatom molecules, the heteronuclear diatom molecule can also be represented by two structures and the actual diatomic molecule are a sort of hybrid.

$$\begin{array}{c} A+B=\frac{1}{2}\left[A^{+x}B^{-x}+A^{-x}B^{+x}\right] \end{array}$$ (1)

Formal charge *x* is a mathematical construct that is useful to understand the behavior of atomic particle. The ability of an atom in heteronuclear diatomic molecules AB to attract bonding electrons to itself is different in a homonuclear molecule A_2_, the process of atom losing or gaining electrons is accompanied by some alteration in hardness and configuration of the particle, the atomic hardness coefficient will have a slight change [2, 12]. If the hardness coefficient of atom A is smaller than that of atom B, it is energetically favorable for the hardness coefficient of atom A increases slightly while that of atom B decreases slightly, atom A hardness is defined as

$$\begin{array}{cc} \eta_{A}^{+} & =\eta_{A}^{+}\left(x\right)\\ & =a_{A}+b_{A}\delta +b_{A}x \end{array}$$ (2)

where *δ* is a minor variation of the atomic hardness coefficient values, *a*_*A*_ and *b*_*A*_ are parameters for the A atom isoclectronic series, the parameters *a* and *b* are different for different atoms and them were obtained for a few atoms in the Letter [1], their exact value depends on the total number of points in the fitting and they are most reliable for the first three rows in the periodic table [13, 14]. The deformation function, *ε*_*A*_(*x*), is given by [1]

$$\begin{array}{cc} \varepsilon_{A}^{+}\left(x\right) & =\frac{\left(a_{A}+b_{A}\delta \right)\upsilon_{A_{0}^{+}}}{b_{A}}\{K_{0}\left(\frac{a_{A}\zeta_{A}}{b_{A}}+\zeta_{A}\delta \right)I_{0}\left(\frac{a_{A}\zeta_{A}}{b_{A}}+\zeta_{A}\delta +\zeta_{A}x\right)\\ & -I_{0}\left(\frac{a_{A}\zeta_{A}}{b_{A}}+\zeta_{A}\delta \right)K_{0}\left(\frac{a_{A}\zeta_{A}}{b_{A}}+\zeta_{A}\delta +\zeta_{A}x\right)\} \end{array}$$ (3)

where $\upsilon_{A_{0}^{+}}$ is the initial velocity of the particle A^+*δ*^, and *ζ*_*A*_ is an undetermined parameter. The Bessel functions *I*_*n*_(*x*) and *K*_*n*_(*x*) are defined in Ref. [15–17] therefore

$$\begin{array}{cc} \frac{d^{2}\varepsilon_{A}^{+}\left(x\right)}{dx^{2}} & =\frac{\left(a_{A}+b_{A}\delta \right){\zeta_{A}}^{2}\upsilon_{A_{0}^{+}}}{b_{A}}\{K_{0}\left(\frac{a_{A}\zeta_{A}}{b_{A}}+\zeta_{A}\delta \right)[I_{2}\left(\frac{a_{A}\zeta_{A}}{b_{A}}+\zeta_{A}\delta +\zeta_{A}x\right)+\\ & \frac{1}{x}I_{1}\left(\frac{a_{A}\zeta_{A}}{b_{A}}+\zeta_{A}\delta +\zeta_{A}x\right)]+I_{0}\left(\frac{a_{A}\zeta_{A}}{b_{A}}+\zeta_{A}\delta \right)\\ & \left[\frac{1}{x}K_{1}\left(\frac{a_{A}\zeta_{A}}{b_{A}}+\zeta_{A}\delta +\zeta_{A}x\right)-K_{2}\left(\frac{a_{A}\zeta_{A}}{b_{A}}+\zeta_{A}\delta +\zeta_{A}x\right)]\right\} \end{array}$$ (4)

taking into account the above functions, the potential energy $E_{A^{+\delta +x}}\left(x\right)$ is defined as

$$\begin{array}{c} E_{A^{+\delta +x}}\left(x\right)=\eta_{A}^{+}\left(x\right)\frac{d^{2}\varepsilon_{A}^{+}\left(x\right)}{dx^{2}}\varepsilon_{A}^{+}\left(x\right) \end{array}$$ (5)

the derivation of $E_{B^{-\delta -x}}\left(x\right)$, $E_{A^{+\delta -x}}\left(x\right)$ and $E_{B^{-\delta +x}}\left(x\right)$ is analogous to $E_{A^{+\delta +x}}\left(x\right)$.

$$\begin{array}{c} E_{B^{-\delta -x}}\left(x\right)=-\eta_{B}^{-}\left(-x\right)\frac{d^{2}\varepsilon_{B}^{-}\left(-x\right)}{dx^{2}}\varepsilon_{B}^{-}\left(-x\right) \end{array}$$ (6)

$$\begin{array}{c} E_{A^{+\delta -x}}\left(x\right)=-\eta_{A}^{-}\left(-x\right)\frac{d^{2}\varepsilon_{A}^{-}\left(-x\right)}{dx^{2}}\varepsilon_{A}^{-}\left(-x\right) \end{array}$$ (7)

$$\begin{array}{c} E_{B^{-\delta +x}}\left(x\right)=\eta_{B}^{+}\left(x\right)\frac{d^{2}\varepsilon_{B}^{+}\left(x\right)}{dx^{2}}\varepsilon_{B}^{+}\left(x\right) \end{array}$$ (8)

An energy formula for the reaction Eq (1) is given

$$\begin{array}{c} E(x)=\frac{1}{2}\left[E_{A^{+\delta +x}}\left(x\right)+E_{B^{-\delta -x}}\left(x\right)+E_{A^{+\delta -x}}\left(x\right)+E_{B^{-\delta +x}}\left(x\right)\right] \end{array}$$ (9)

In most cases atoms will be unstable on their own and they have to bond with others and exchange charge for stability [18]. The energy of a system of homonuclear diatomic molecules depends on the exchange charge between them and the potential energy minimum occurs at *ζ* = *ζ*_0_. The stable atoms are found to obey the equation of state given by [1]

$$\begin{array}{c} \frac{a\zeta_{0}}{b}≐2.6018 \end{array}$$ (10)

In the isoelectronic series like this, in which the neutral atom is isoelectronic with each of the following ions, the hydrogen-like series has more accurate fitting parameters of ionization energy.

$$\begin{array}{c} \frac{a_{H}{\left(\zeta_{0}\right)}_{H}}{b_{H}}=2.60182179 \end{array}$$ (11)

This value(2.60182179) can then be used in all subsequent calculations. When the atom is in a different environment, the deformation parameters and the hardness coefficient should be changed [19, 20]. Using Eq (11), it is easy to get the equations of state of different atoms, so that the parameters *ζ*_*A*_ and *ζ*_*B*_ can be given by

$$\begin{array}{c} \zeta_{A}\left(\frac{a_{A}}{b_{A}}+\delta \right)=2.60182179 \end{array}$$ (12)

$$\begin{array}{c} \zeta_{B}\left(\frac{a_{B}}{b_{B}}-\delta \right)=2.60182179 \end{array}$$ (13)

Then, combine Eqs (12), (13) and (9), eliminate *ζ*_*A*_ and *ζ*_*B*_. In this case, the problem is to find the solution of an equation with a variable *x* and a minor variation values *δ* of the atomic hardness coefficient.

$$\begin{array}{c} E=E(x,\delta ) \end{array}$$ (14)

### 2. The equation of the Cassini oval

The initial velocity, $\upsilon_{A_{0}^{+}}$ and $\upsilon_{B_{0}^{-}}$, in Eq (14) can be determined by the Cassini oval model below.

In homonuclear diatomic molecules, the initial reaction velocity depends on the ground state electron configuration of the atom and an empirical formula [1] is proposed to estimate the *υ*_0_

$$\begin{array}{c} \upsilon_{0}=2^{\ell /2}\cdot \left(N-\ell \right)\cdot \frac{J^{1/2}}{P^{1/2}} \end{array}$$ (15)

where

$$\begin{array}{cc} J & ={\left(\frac{d+V}{2}\right)}^{1/2}+{\left(dV\right)}^{3-N}+\left(d-1\right)\{\frac{2d}{d+V}\\ & +\frac{{\left(-1\right)}^{V}d}{NV}\text{cot}\left(\frac{(2V-3)\pi }{2N}\right)\}+\left(\frac{d-V}{4}\right)\text{tan}\left(\frac{5\pi }{2N}\right) \end{array}$$ (16)

and

$$\begin{array}{c} P=2^{\left(\ell -1)\Gamma (N\right)}N^{(1-\ell )(N+1)/2}\left(N+2^{N-1}\right) \end{array}$$ (17)

Γ() is the Gamma function, *N* is the principal quantum numbers, *ℓ* is the azimuthal quantum numbers, *d* are unpaired electrons of the ground state electron outside the nucleus and *V* are empty slots in it’s valence electron orbits.

**Fig 1:**
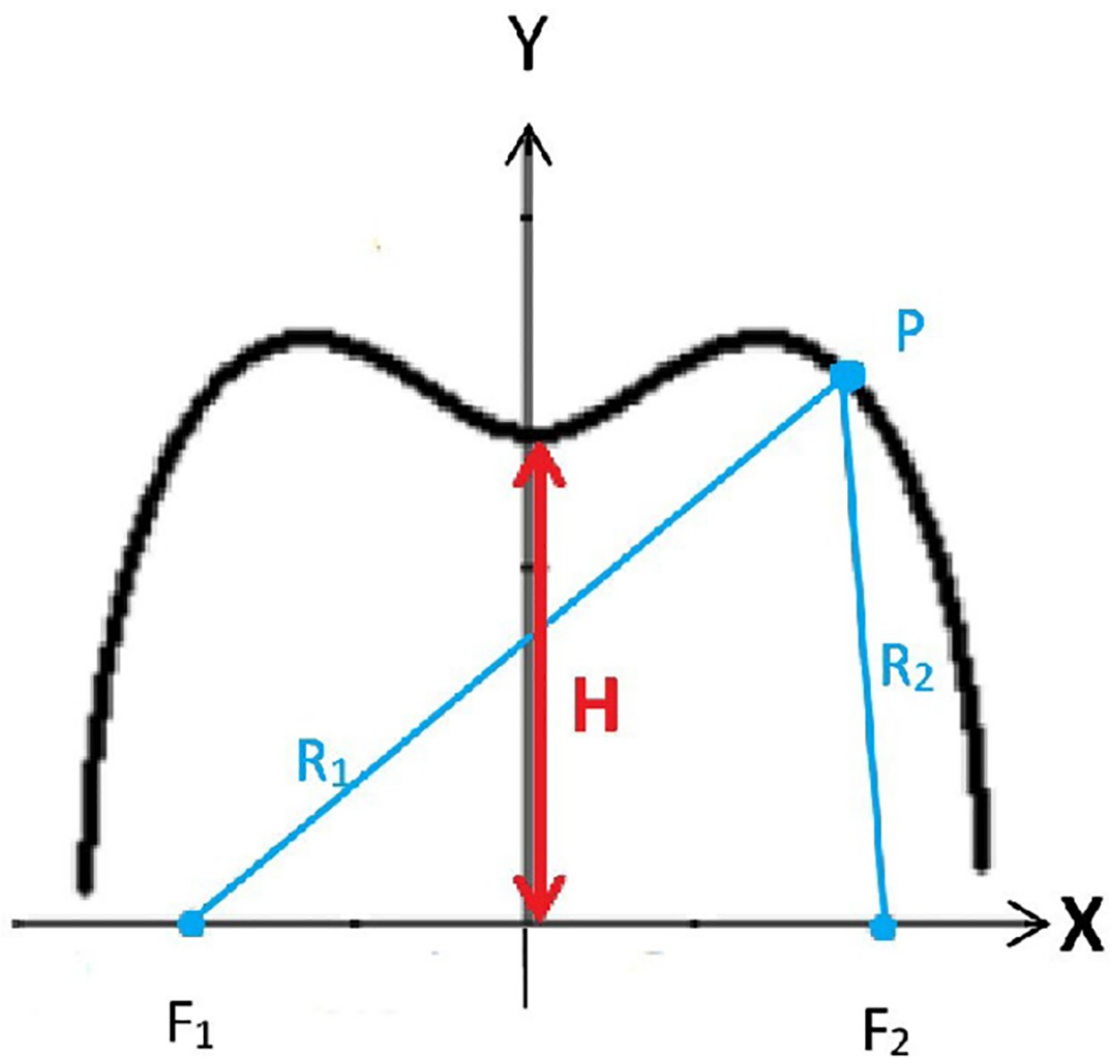
A semi-Cassini oval with foci F1 and F2 on the x-axis.

In heteronuclear diatomic molecules, the initial velocity of an atomic particle is related not only to the structure of electrons that an atom possesses but also to the hardness and shape of atoms. The initial velocity of the atomic particle can be expressed as

$$\begin{array}{c} \upsilon_{A_{0}^{+}}=\alpha \upsilon_{A_{0}} \end{array}$$ (18)

$$\begin{array}{c} \upsilon_{B_{0}^{-}}=\beta \upsilon_{B_{0}} \end{array}$$ (19)

where *α* and *β* are state variables of molecules, $\upsilon_{A_{0}}$ and $\upsilon_{B_{0}}$ are the initial velocity of deformation of the atoms A and B in homonuclear diatomic molecules, respetively. The Cassini ovals are defined by the equation

$$\begin{array}{c} R_{1}R_{2}={\left(\frac{\alpha }{\beta }\right)}^{2} \end{array}$$ (20)

where *R*_1_ and *R*_2_ denote the ranges *F*_1_ to *P* and *P* to *F*_2_ respectively as in Fig 1. In the Cartesian plane, the origin is represented by an ordered pair (0, 0), then the foci will have the coordinates $F_{1}\left(-\beta^{1/2}\upsilon_{B_{0}},0\right)$ and $F_{2}\left(\alpha^{1/2}\upsilon_{A_{0}},0\right)$, the Cassini ovals have the following equation

$$\begin{array}{c} {}[{\left(x-\alpha^{1/2}\upsilon_{A_{0}}\right)}^{2}+y^{2}][{\left(x+\beta^{1/2}\upsilon_{B_{0}}\right)}^{2}+y^{2}]={\left(\frac{\alpha }{\beta }\right)}^{4} \end{array}$$ (21)

The top half of a Cassini oval has the equation

$$\begin{array}{cc} y & =2^{\frac{-1}{2}}\{-{\left(x-\alpha^{1/2}\upsilon_{A_{0}}\right)}^{2}-{\left(x+\beta^{1/2}\upsilon_{B_{0}}\right)}^{2}+\\ & {\left[{\left[{\left(x-\alpha^{1/2}\upsilon_{A_{0}}\right)}^{2}-{\left(x+\beta^{1/2}\upsilon_{B_{0}}\right)}^{2}\right]}^{2}+4{\left(\frac{\alpha }{\beta }\right)}^{4}\right]}^{\frac{1}{2}}{\}}^{\frac{1}{2}} \end{array}$$ (22)

The Cassini shape depends on the state variables *α* and *β*, the shape factor Φ is defined as

$$\begin{array}{c} \Phi =\frac{2H}{\alpha^{1/2}\upsilon_{A_{0}}+\beta^{1/2}\upsilon_{B_{0}}} \end{array}$$ (23)

where H is a local minimum (or maximum) value of the Eq (22) at $x=\alpha^{1/2}\upsilon_{A_{0}}-\beta^{1/2}\upsilon_{B_{0}}$.

Choosing an appropriate value of Φ, a series of *β* values corresponding to *α* values can be obtained from the Eq (22).

### 2.3. State function

After determining the initial velocity,$\upsilon_{A_{0}^{+}}$ and $\upsilon_{B_{0}^{-}}$ in Eq (14), *δ* and the potential energy can be given by a graphical method [1]. Analyzing large amount of data originating from this model, it is found that the state function of the molecule can be expressed by the following equation

$$\begin{array}{c} \psi =\frac{\alpha }{\beta }\left(\delta^{1/2}-\delta \Phi^{2}\right) \end{array}$$ (24)

Plotting *ψ* against *α*, and the graph is a parabola that opens upward, the vertex occurs at the lowest point on the graph. It is necessary to make the graph have a vertical axis of symmetry by adjusting the model parameters, the equation for the axis of symmetry is the *α*-value of the vertex coordinates and the parameters should be most accurately calculated so as to maximize the precision of the model.

**Fig 2:**
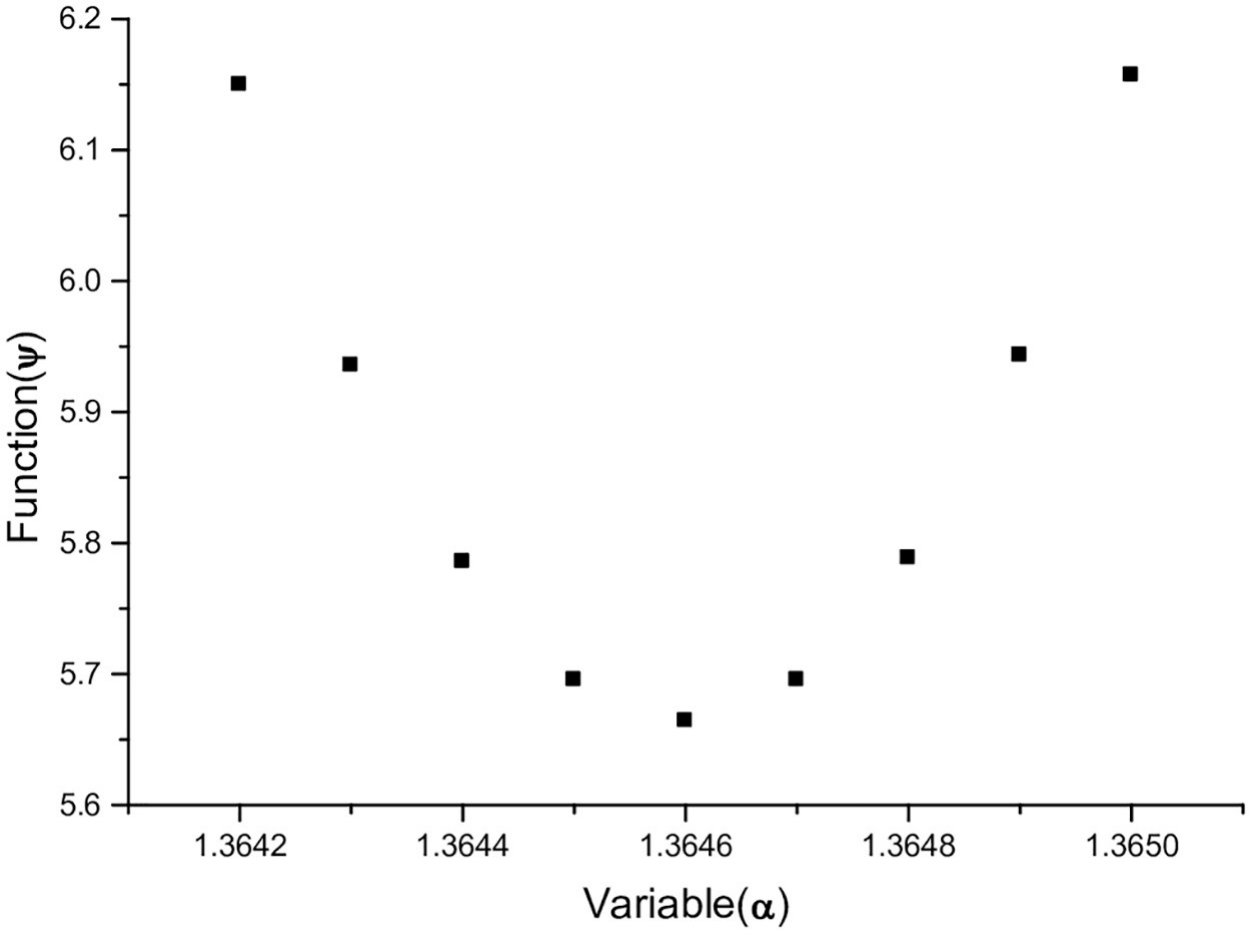
Φ = 0.537478822300558, the graph of the function *ψ*(*α*) is a parabola.

This is an example of *HO* molecule. It is given that at *α* = 1.3646, finding the Φ value can be a process of trial and error, until the function *ψ* attains its minimum value of a parabola at Φ = 0.537478822300558; using the same Φ to calculate the function *ψ*(*α*) on the interval [1.3642, 1.3650]. These *ψ*(*α*) values have 14 significant digits whose first 9 digits are the same. In order to better distinguish the vertical axis spacing of graphs, the last 5 digits with a length of 14 significant digits are extracted as a new value to plot the function *ψ*(*α*), this is shown in Fig 2.

In Fig 2, this model only needs the data of the vertex of the parabola. For the function *ψ*(*α*) of *HO* molecule, a set of data of the lowest point of the parabola is given in Table 1.

**Table 1:** A set of data of the lowest point of the parabola.

parameter	value
*α*	1.3646000000000
*β*	0.739481316335810
Φ	0.537478822300558
δ	0.0013069029483525
*ψ*	0.066014668056642

The same types of function value can be obtained by changing the value of Φ, and the potential energy can be calculated by using the lowest point data of the graph.

### 4. Critical point

**Fig 3:**
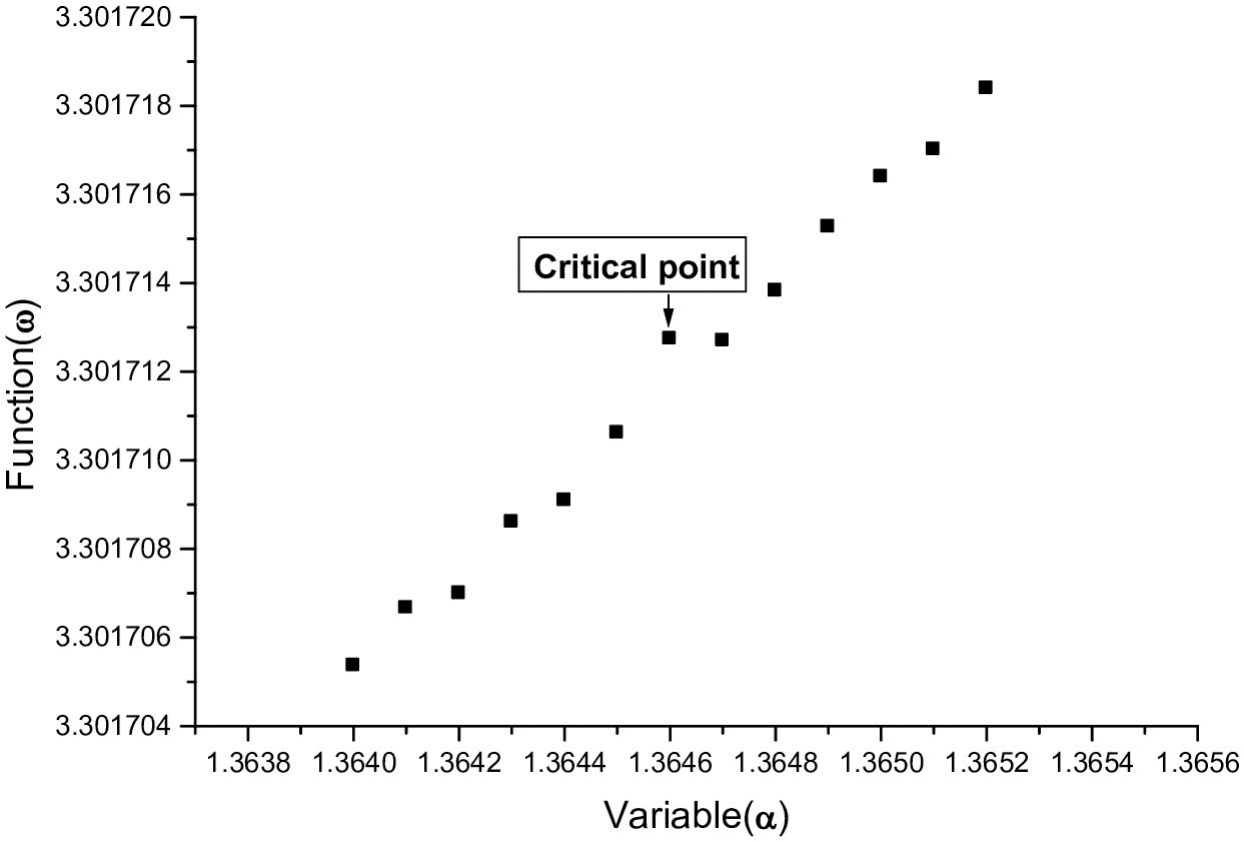
*μ* = 1.432, the graph of *ω*(*α*) has a critical point at *α* = 1.3646.

Using the same method as the Letter [1], the depth of potential well *D* and *δ* can be determined accurately by graphing. For each value of Φ, the vertex coordinates of the function *ψ*(*α*) are obtained by trial and error. The analysis of vertex data shows that as the increase of Φ value, the value of *D* decreases. A new function is introduced as a tool for analyzing molecular stability

$$\begin{array}{c} \omega =\frac{D\Phi^{\mu }}{{\left(\alpha \upsilon_{A_{0}}+\beta \upsilon_{B_{0}}\right)}^{2}} \end{array}$$ (25)

where *μ* is disturbance factor, the *D*, *α* and *β* correspond to the lowest point of the graph of the function *ψ*(*α*). For *HO* molecule, when *μ* ⩽ 1.431, the function *ω*(*α*) increases on the interval [1.3640, 1.3652]; and *μ* = *μ*_1_ = 1.432, the graph of *ω*(*α*) has a critical point at *α* = 1.3646. For *ω*(*α*) whose graph is shown in Fig 3.

**Fig 4:**
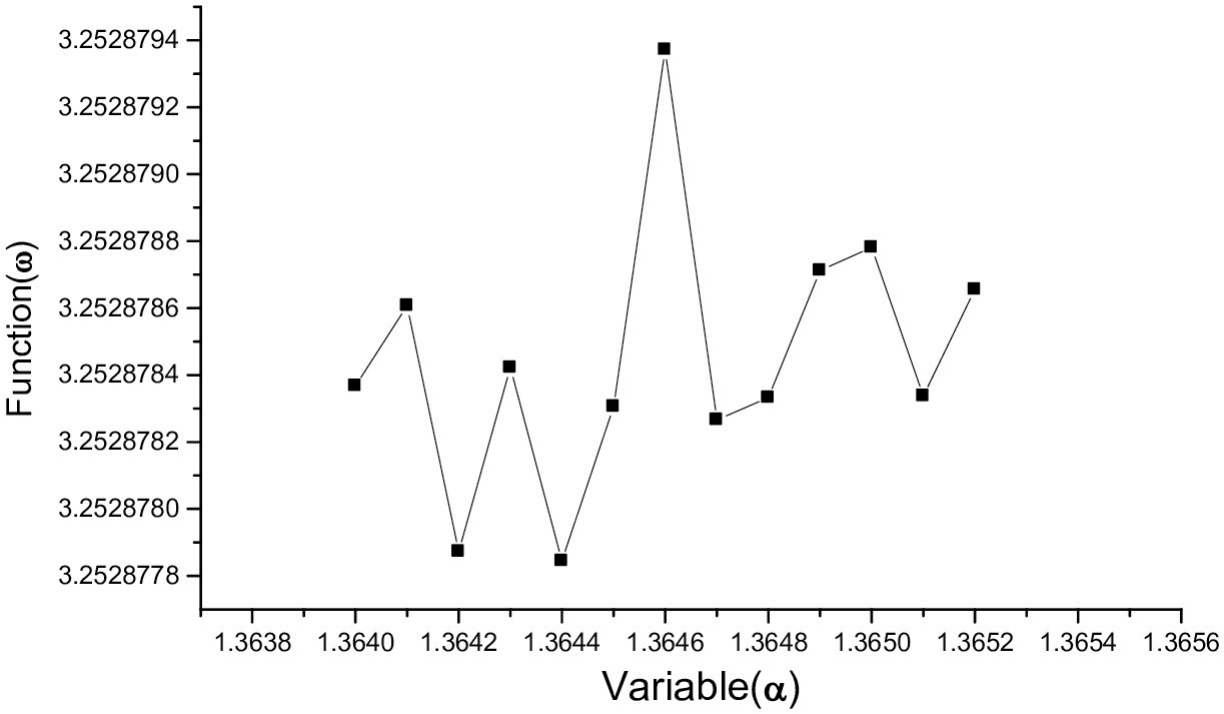
*μ* = 1.456, the graph of *ω*(*α*) on the interval [1.3640, 1.3652].

When *ω*(*α*) changes slowly as *μ* increases from 1.431 to 1.456 so the graph becomes irregular, jagged waveform on the interval [1.3640, 1.3652], *ω*(*α*) whose graph is shown in Fig 4.

**Fig 5:**
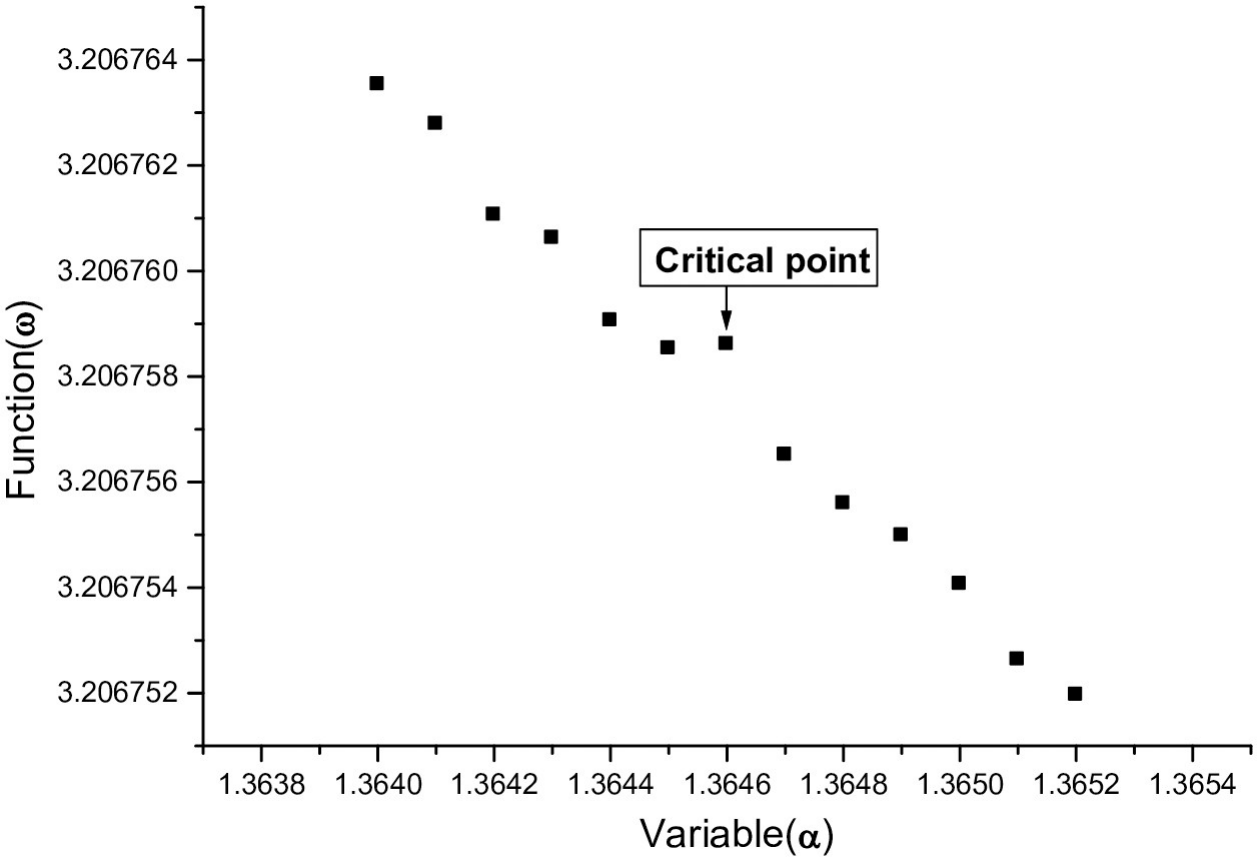
*μ* = 1.479, the graph of *ω*(*α*) has a critical point at *α* = 1.3646.

As *μ* continuing to increase and *μ* = *μ*_2_ = 1.479, the graph of *ω*(*α*) has only a single critical points at *α* = 1.3646 again; then the critical points disappear and the function *ω*(*α*) decreases on the interval [1.3640, 1.3652] for *μ* > 1.479, this is given in Fig 5.

In Figs 3 and 5 above, note that the critical points on the curve of the function *ω*(*α*) have the same *α*-coordinate (*α* = 1.3646), the model determines that the two critical points have the same value of *α* and the results can be reproduced by using the same method to calculate other molecules.

Expanding the scope of data analysis, the results obtained are consistent with the above discussions

$$\begin{array}{ll} \mu <\mu_{1}(1.432), & \omega (\alpha )\;\text{is}\;\text{increasing}\;\text{on}\;(1.3528,1.3841)\\ \mu_{1}⩽\mu ⩽\mu_{2}, & \omega (\alpha )\;\text{is}\;\text{fluctuating}\;\text{on}\;(1.3528,1.3841)\\ \mu >\mu_{2}(1.479), & \omega (\alpha )\;\text{is}\;\text{decreasing}\;\text{on}\;(1.3528,1.3841) \end{array}$$

## Results

In this investigation, one wants to explore the locus created by the vertices of the parabola functions *ψ*(*α*), each graph of function *ψ*(*α*) must have a vertical axis of symmetry. Once the locus of the vertices of the family of parabolas, then the molecular potential energy can be determined. At *μ* = *μ*_1_ and *μ* = *μ*_2_, the critical point of the function *ω*(*α*) have the same *α*-coordinate and the results of these molecules are consistent with those of HO molecule, the related parameter values of some molecules are shown in Table 2.

**Table 2:** The parameters of some molecules function *ω* at the critical point.

A^+*δ*^B^−*δ*^	Φ	*μ*_1_	*α*	*D*
	*δ*	*μ*_2_	*β*	
HLi	0.80084358057894	1.242	1.02110000000000	48.611051630227
	8.5398133002360 × 10^−4^	1.277	0.664593779607782	
HF	0.39771371997290	1.096	1.598700000000000	116.628240125172
	5.3338564273950 × 10^−4^	1.409	1.055290565947800	
HO	0.537478822300558	1.432	1.364600000000000	87.727364974559
	1.3069029483525 × 10^−3^	1.479	0.739481316335810	
HS	0.37889316682936	1.486	1.251300000000000	72.327432394782
	7.7631958654476 × 10^−4^	1.809	0.695779900417062	
HCl	0.29872973188655	1.136	1.391400000000000	88.170244934352
	4.5433194765157 × 10^−4^	2.028	0.866877878288910	
FCl	0.84959818830720	1.220	1.63740000000000	53.220214733972
	1.1830861584273 × 10^−2^	1.224	0.915452892542364	
OS	0.70169721776072	1.060	1.396700000000000	106.06810336619
	3.3659893881879 × 10^−3^	1.086	0.594773134971361	
OF	0.88302810235504	0.550	0.899600000000000	44.887071995685
	1.3485177893819 × 10^−3^	0.553	0.539843627069544	
OCl	0.59308532370214	1.088	1.014400000000000	54.977888277740
	3.0677072532017 × 10^−3^	1.095	0.577504922783953	
SCl	0.79144936398030	0.619	1.012550000000000	49.648454001822
	2.7035852713700 × 10^−3^	0.629	0.494349755251032	
NO	1.10190606039122	0.239	1.124000000000000	129.58047019764
	7.133447109443 × 10^−5^	0.600	0.384343072022964	
CO	0.79269899567280	0.598	1.82140000000000	219.55929359009
	1.3704258094196 × 10^−3^	0.634	0.63184419913609	
CS	0.55356948381750	0.947	1.506600000000000	145.91777971069
	1.7762867876860 × 10^−3^	1.029	0.597071856106433	
FS	1.04683120615265	0.927	1.796100000000000	69.997410539025
	1.1482197556274 × 10^−2^	0.929	0.771507111268300	

Based on the theoretical and experimental values of hydrogen molecules, the potential energy formula is given by

$$\begin{array}{c} E^{cal}=\frac{D_{AB}E_{H_{2}}^{exp}}{D_{H_{2}}} \end{array}$$ (26)

where $D_{H_{2}}$ is calculated well depth of hydrogen molecule, $D_{H_{2}}=89.1287$; *D*_*AB*_ is well depth data for each of the 14 molecules in Table 2; $E_{H_{2}}^{exp}$ is experimental dissociation energy of hydrogen molecule, $E_{H_{2}}^{exp}=435.7799kJ/mol$. Table 3 shows the results of Eq (26), and relevant experimental data are collected from scientific literature [21].

**Table 3:** The data of experimental and calculated energies for some molecules.

A^+*δ*^B^−*δ*^	*E*^*cal*^(*kJ*/*mol*)	*E*^*exp*^(*kJ*/*mol*)
HLi	237.6756	238.039±0.006
HF	570.2343	569.680±0.011
HO	428.9283	429.91±0.29
HS	353.6329	353.57±0.30
HCl	431.0937	431.361±0.013
FCl	260.2411	260.83
OS	518.6023	517.90±0.05
OF	219.4678	220
OCl	268.8052	267.47±0.08
SCl	242.7478	241.8
NO	633.5621	631.62±0.18
CO	1073.4985	1076.38±0.67
CS	713.4406	713.3±1.2
FS	342.2406	343.5±6.7

Analysis results show that the correlation coefficient between the *E*^*cal*^ and *E*^*exp*^ is 0.99999, *P* < 0.0001. The calculated bond energy is in good agreement with existing experimental data, and the bond dissociation energy is related to the depth of the potential well.

The hardness coefficients of heteronuclear atoms are close to each other in the process of combining to form molecules, and the atoms try to obtain more stable configuration. If the hardness coefficient of atom A is less than the atom B, the critical phenomena determine AB molecular structure: not B^+*δ*^A^−*δ*^ but A^+*δ*^B^−*δ*^, the hardness coefficient of atom A increases by *δ*, while that of atom B decreases by *δ*, that is, a quantitative increase in the hardness coefficient of one atom in a molecule is always accompanied by an equal decrease in the hardness coefficient of another atom in the molecule. This model supports a rule between the hardness coefficients of different atoms, conservation of hardness coefficient, only in this way can the molecular potential energy be calculated with the derived equations, it provides a new way for finding the equilibrium of hardness coefficient between two heteronuclear atoms.

The special feature of this model is that the molecular potential energy is as a function of the exchange charge, which is fundamentally different from the work of other scholars. In 2021, Judith P. and Maikel Y. [22] present a historical review of about 50 potential energy functions for diatomic systems, which have been proposed from 1920 to 2020. In this review, the functional forms used to analytically represent potential energy as a function of interatomic distance for diatomic systems is presented.

## Conclusion

Geometry has always played a key role in the formulation and understanding of physical theories [23]. In this paper, it is assumed that there is some relationship between the heteronuclear bimolecular state function and Cassini oval, elliptical or hyperbolas. In the preliminary analysis of the molecular potential energy, hardness coefficient and other data, it is possible to establish a new molecular state equation by Cassini oval model. So this paper focus on exploring the relationship between the molecular state function and the Cassini oval shape. The study shows that the dynamic Cassini oval can be used to describe and analyze the changes of molecular state functions. Then using ellipse or hyperbolas in the same program not produce results similar to Cassini ellipse, which means that ellipse or hyperbolas can not be used to establish molecular state functions.

In this paper, the conjecture of conservation of hardness coefficient is the foci of this model. Based on this idea, a formula for calculating molecular bond energy is derived by Newton’s mechanical methods. An important finding is that the state variable of the molecular function corresponds to the shape of the Cassini oval, the principle of determining the parameters is that the graph of the state function must be a parabola with a vertical axis of symmetry. It is an interesting coincidence that the critical point appears and disappears in the same place. The calculated potential energy at the critical point is consistent with the experimental dissociation energy of molecules, which proves the rationality of model and hypothesis. Extending the Cassini oval geometry, the model can be used to calculate larger and more complex molecular systems, which makes it possible to calculate the deformation stress and electronegativity of atoms in molecules, and provides a new method for evaluating molecular hardness and designing new materials. The conservation of hardness coefficient may be a basic law of nature. The symmetry of molecular functions and the conservation of hardness coefficients are closely related to the lowest energy state of molecules, and the potential deep nature and significance need to be explored. Further research is expected to verify this hypothesis from a much greater and broader scope. This model successfully applied the Newton mechanics method to calculate the potential energy of heteronuclear diatomic molecules, and it is possible to open up a simpler calculation than wave mechanics.

## Supporting information

S1 File Description of calculation method. This instruction manual describes the calculation program used in this paper, and describes the meaning of the symbols in the calculation formula.
(PDF)

S1 Appendix Potential energy formula. Formula for calculating the potential energy of the HO molecules.
(MCD)

S2 Appendix Drawing data 1. The data of drawing the state function diagram of HO molecules.
(MCD)

S3 Appendix Drawing data 2. The data of drawing the critical point diagram of HO molecules.
(MCD)
