# Supplementary material for: Cassini-oval description of atomic binding: New insights into the quantitative relationship between hardness coefficient and bond energy energy
Source: PLoS One. 2024 May 16;19(5):e0303311. doi: 10.1371/journal.pone.0303311 (PMC11098323; doi:10.1371/journal.pone.0303311)
Supplement: S1 File — This instruction manual describes the calculation program used in this paper, and describes the meaning of the symbols in the calculation formula. (PDF) [file pone.0303311.s001.pdf]

Description of calculation method

The calculation program used in this work is MathCAD2001

S2 Appendix. Potential Energy Formula.

Relevant symbol in Molecular (HO) Potential Energy Formula

The a, b value of an A atom is expressed in the formula as:

a={A}; b={b}

The a, b value of an B atom is expressed in the formula as:

a={U}; b={O}

a minor variation of the atomic hardness coefficient values={c}

Deformation coefficients of neutral A atoms={k}

Deformation coefficients of neutral B atoms={g}

gamma constant={r}

The rate of deformation of A atoms={P}

The rate of deformation of B atoms={J}

Constants in Constraint Rules={a}

Potential Energy={p(x)}

S3 Appendix. Drawing data 1

The data of drawing the state function diagram of HO molecules. {the DATA of the lowest point of the parabola parameters }

S4 Appendix. Drawing data 2.

The data of drawing the critical point diagram of HO molecules. { the DATA of ω(α) }
